# Control of finite critical behaviour in a small-scale social system

**DOI:** 10.1038/ncomms14301

**Published:** 2017-02-10

**Authors:** Bryan C. Daniels, David C. Krakauer, Jessica C. Flack

**Affiliations:** 1 ASU-SFI Center for Biosocial Complex Systems, Arizona State University, Tempe, Arizona 85287, USA; 2 Santa Fe Institute, Santa Fe, New Mexico 87501, USA

## Abstract

Many adaptive systems sit near a tipping or critical point. For systems near a critical point small changes to component behaviour can induce large-scale changes in aggregate structure and function. Criticality can be adaptive when the environment is changing, but entails reduced robustness through sensitivity. This tradeoff can be resolved when criticality can be tuned. We address the control of finite measures of criticality using data on fight sizes from an animal society model system (*Macaca nemestrina*, *n*=48). We find that a heterogeneous, socially organized system, like homogeneous, spatial systems (flocks and schools), sits near a critical point; the contributions individuals make to collective phenomena can be quantified; there is heterogeneity in these contributions; and distance from the critical point (DFC) can be controlled through biologically plausible mechanisms exploiting heterogeneity. We propose two alternative hypotheses for why a system decreases the distance from the critical point.

## Abstract (web summary)

Proximity to criticality can be advantageous under changing conditions, but it also entails reduced robustness. Here, the authors analyse fight sizes in a macaque society and find not only that it sits near criticality, but also that the distance from the critical point is tunable through adjustment of individual behaviour and social conflict management.

Over the last decade new technologies for making large numbers of fine-grained measurements have led to the surprising discovery that many biological systems sit near a critical point^1^,^2^. When a system is near the critical point small changes to component behaviour can induce large-scale changes in aggregate structure and function^3^,^4^,^5^. Examples of systems in which this behaviour has been observed include networks of neurons^6^, ant groups cooperatively carrying a load^5^ and animal groups forming flocks and schools^7^,^8^,^9^,^10^. Accounting for criticality remains a challenge as sensitivity to perturbation suggests a lack of robustness. Furthermore, change induced by perturbation may not be adaptive. Complicating matters further, critical phenomena can result from history-dependent stochastic processes^11^. A question central to distinguishing among these conflicting views of criticality is to what degree criticality can be controlled by the components of the system^2^.

We address the control of criticality using data on fight sizes from an animal society model system (*Macaca nemestrina*, *n*=48). Like many biological systems, it contains relatively few individuals, which means we cannot use standard physics measures to define critical behaviour. Instead, we introduce a two-component operational definition of criticality for finite systems that uses (1) the Fisher information to capture sensitivity across scales and (2) a measure of collective instability. We ask whether conflict behaviour in the model system is critical using empirically grounded equilibrium (maximum entropy) and dynamic (branching process) models of the monkeys' fight-joining behaviour. We find that the system does sit near a critical point, and we quantify this distance in terms of the number of individuals that would need to be perturbed to reach the point of maximal sensitivity and instability.

## Results

### Study system and modelling framework

Our analysis begins with a time series of fights from a large, captive pigtailed macaque group collected over multiple observation periods during a four month period (see ‘Methods' section). The data consist of a series of binary fight participation vectors **x** of length *n*. For each vector an individual is assigned a ‘1' if it participated in that fight and a ‘0' if it did not (see ‘Methods' section).

To study criticality we modify tools from statistical mechanics. These tools are best deployed when the study system can be described using a simple, tractable and well-understood modelling framework like a spin-glass or branching process. Hence our first task is to assess whether these models are empirically justified descriptions of our study system. To assess this, we ask whether our data are consistent with any of three basic fight-joining models: (1) decisions to join fights are independent, (2) decisions to join are correlated (equilibrium model) and (3) decisions to join fights are strategic with correlations resulting from one individual joining in response to a second individual joining (dynamical branching process)^12^. We evaluate these models by determining how effectively each recovers a key social feature—the distribution of fight sizes *s* (ref. 13)—when parameterized by the empirical data.

The independent model simply takes into account the individual fight-joining frequencies 〈*x*_*i*_〉 (see ‘Independent model inference' in Methods).

The correlated decision-making model (see ‘Model descriptions and justification' in Methods) is an equilibrium maximum entropy model that fits all pair-wise correlations and corresponds to a spin-glass model^14^. The word equilibrium is used here to indicate that interactions governing fights do not change over time (stationarity) and that time within fights does not play an explicit role (simultaneity). The resulting probability distribution over possible fights has relative negative log likelihood


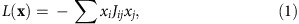


where the coefficients *J*_*ij*_ are numerically fit to match individual and pairwise frequencies 〈*x*_*i*_〉 and 〈*x*_*i*_*x*_*j*_〉 (see ‘Pairwise maximum entropy model inference' in Methods).

In the dynamical branching process (see ‘Model descriptions and justification' in Methods), the dominant causes of conflict are temporal pairwise interactions—an individual joins the current fight with some finite probability only when it sees another individual join. Fight initiation is assumed to occur at a slower timescale, when a random individual becomes aggressive. Individuals join the fight by receiving aggression from or initiating aggression against an individual already in the fight. Parameters include individual initiation parameters *p*_0*i*_, each denoting the relative probability that individual *i* begins a fight, and conditional redirection parameters *p*_*ij*_, each denoting the probability that *j* joins a fight due to *i* having just joined. We consider only the relative ordering of individuals joining fights, not direct interactions, and estimate parameters by fitting conditional frequencies *P*_*ij*_—the frequency of seeing individual *j* at any later time in a fight sequence given that *i* appeared first (see ‘Branching process inference' in Methods).

**Figure 1:**
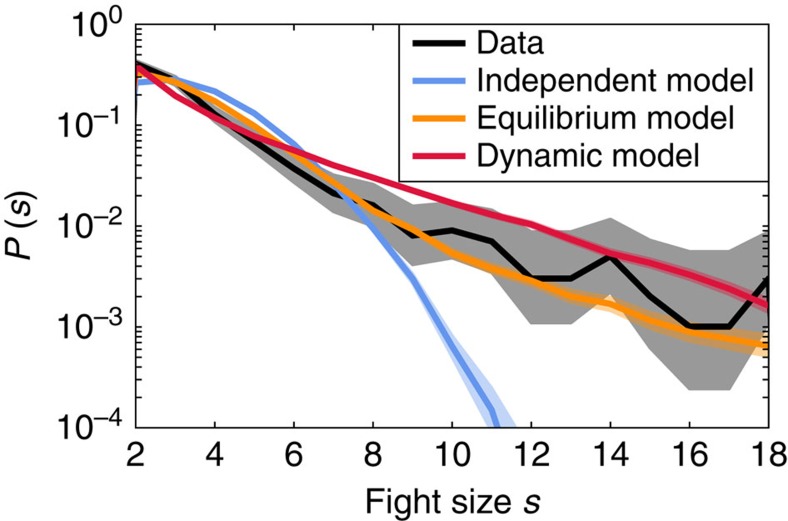
Testing fit of fight-joining processes to individual-level data. Models that include social correlations (the equilibrium maximum entropy and dynamic branching process models) can reproduce the relatively long tail of the observed distribution of fight sizes, whereas assuming independent fight-joining events (the independent model) cannot. Shaded regions indicate 95% confidence intervals.

We test the performance of each model through its ability to predict the distribution of fight sizes (Fig. 1) and correlations up to 3rd order (Supplementary Fig. 7, Supplementary Note 2). We rule out the independent model (Supplementary Note 2). We find the empirically parameterized maximum entropy (as in prior work^14^) and branching process models recover the observed distribution of fight sizes, indicating that these models are mechanistically consistent with the data. We can now use these empirically parameterized models to investigate whether the system is near critical.

### Criticality as sensitivity and instability

Critical behaviour is closely linked to what in physics is called a phase transition. A phase transition (Supplementary Note 3) can be thought of as a point at which the system becomes infinitely sensitive to perturbations when the number of individuals becomes infinite. This diverging sensitivity is caused by a collective instability, meaning that perturbations to individuals spread to change the behaviour of the entire system (Supplementary Note 3, details on collective instability).

Phase transitions are typically identified by examining the asymptotic behaviour of the infinite limit (for a review of definitions, see ref. 2). Social systems like the one studied here are, however, not large enough to be well described by the infinite limit. In addition, it is natural in these systems to focus on the effects caused by individual components, which is different from the typical case in physics, where tuning parameters are external and applied at a macroscopic level. These differences make the limit of an infinite number of components awkward in social systems. For these reasons we move to an operational definition of critical points that captures the core idea of criticality in infinite systems appropriate in the finite case. This definition takes into account two quantities—sensitivity and collective instability.

We define sensitivity as the derivative of the average fight size with respect to an individual's agitation, averaged over individuals. In the equilibrium model, the control parameter associated with the average fight size 〈*s*〉 is an external ‘field' *h*_ext_, which we interpret as uniformly increasing each individual's agitation, making it more likely to become involved in fights. Adding an external field to equation (1), we have 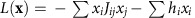, where *h*_*i*_=*h*_ext_ for all *i*. The corresponding sensitivity is equal to the susceptibility *χ*:


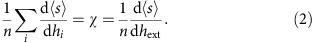


A sharp peak in susceptibility is one indicator that a phase transition may be present in the corresponding infinite system. Typically this is verified by measuring the growth of the peak with increasing system size^15^. Instead, for a system with a fixed number of individuals, we will later verify that the peak is caused by an instability that would lead to such growth.

**Figure 2:**
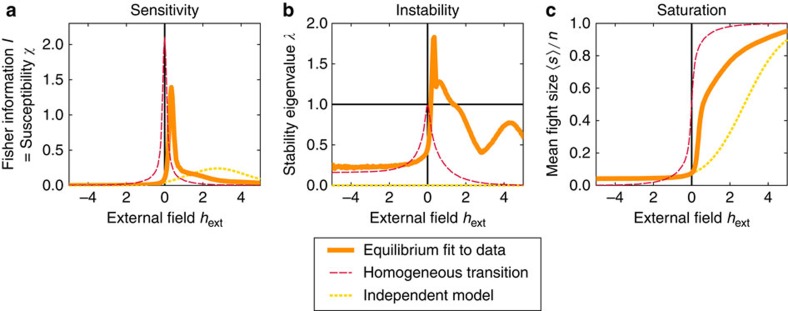
The system is near a sensitive and unstable region of parameter space. (**a**) The susceptibility *χ*, measuring the sensitivity of average fight size to an external perturbation, has a peak near the fit parameters (at *h*_ext_=0). The susceptibility is also equal to the Fisher information *F*(*h*_ext_), describing how quickly the distribution over fights becomes distinguishable as *h*_ext_ is varied. (**b**) Instability of the lowest order mean-field solution is indicated by the eigenvalue *λ* becoming larger than 1. Yellow dashed lines indicate a system of the same size with independent individuals, and red dashed lines indicate a homogeneous system of the same size tuned to be marginally unstable at *h*_ext_=0. (**c**) The transition is associated with large changes in mean fight size.

Results for the equilibrium model are shown in Fig. 2a. For fit parameters (*h*_ext_=0), there is increased sensitivity compared with a non-interacting model with the same mean fight size, meaning that an amplification process is occurring that makes changes to patterns of aggression at the individual level ‘visible' at the global system level. This amplification process cannot be attributed to external events as these data were collected in a captive setting in which such disturbances were minimized (see ‘Model descriptions and justification' in Methods).

The susceptibility can also be interpreted as a Fisher information, an information theoretic quantity that describes how sensitive a distribution is to the parameters that describe it (Supplementary Note 5). A large *χ* implies faster learning: large susceptibility means that aggregate level statistics are informative about conflict dynamics at the individual level^16^,^17^.

Analogously to the susceptibility in the equilibrium model, we can define sensitivity in the dynamic model as how quickly fight sizes grow following perturbation of redirection probabilities *p*_*ij*_:


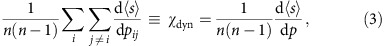


where *p* adds probability uniformly to all redirection probabilities.

We define collective instability using perturbation theory. This is straightforward in the branching model: when the system is perturbed by one individual becoming active, the average number of other individuals triggered in the next time step, *R*_0_, indicates whether perturbations are amplified or decay. If *R*_0_ is above 1, perturbations grow exponentially and the peaceful state of the system is unstable. In our dynamic model, this linear stability of the peaceful state is indicated by the largest eigenvalue *R*_0_ of the redirection probability matrix *p*_*ij*_ (corresponding to the least stable mode of the system).

In the case of the equilibrium model, an analogous quantity *λ* measures the stability of the mean-field solution, corresponding to the eigenvalue with largest magnitude of the matrix *M*_*ij*_=−2(1–*δ*_*ij*_)*J*_*ij*_〈*x*_*i*_〉(1–〈*x*_*i*_〉) (see Supplementary Note 3, details on collective instability). Results for the equilibrium model stability at increasing *h*_ext_ are shown in Fig. 2b.

In each model, the stability factor (*R*_0_ or *λ*) becoming >1 indicates a true phase transition in the case when the number of individuals in the system becomes infinite. In a finite system, the stability, like the sensitivity, is a useful measure in its own right, one that we use as an indicator of whether a peak in sensitivity is caused by a collective instability.

### Distance from criticality

Figure 2 suggests that the system is near a transition. Comparing to a simple homogeneous system of the same size that is poised directly at instability (red dashed lines in Fig. 2), we see in the inferred model a similar peak in sensitivity, but one that is shifted to occur when the system is exposed to a field that increases overall agitation. The magnitude of the field necessary to reach the peak defines a distance from the transition, but one that is measured in units that are not easily related to biology.

To quantify in a biologically meaningful way how much agitation is required to move the system towards the critical point, we measure how sensitivity and stability vary as we force-through parameterized-simulation (Supplementary Note 3) some number of individuals to fight continuously (while allowing the system to consist only of the non-forced individuals). The number of individuals required to reach a peak in sensitivity corresponding to a collective instability (Supplementary Note 3) is a measure of the distance of the system from the critical point (DFC). Beyond this peak lies saturation and decreasing sensitivity.

In the equilibrium model forcing is accomplished by removing the forced individuals and adding their interaction terms to the fields acting on the remaining individuals: the new fields are given by 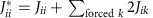. In the dynamic model this is accomplished by explicitly including the forced individuals at each time step in the simulation but only recording the behaviour of the remaining individuals. The change in sensitivity caused by forcing each individual also provides ‘sensitivity scores' that quantify the extent to which each individual brings the system closer to criticality.

**Figure 3:**
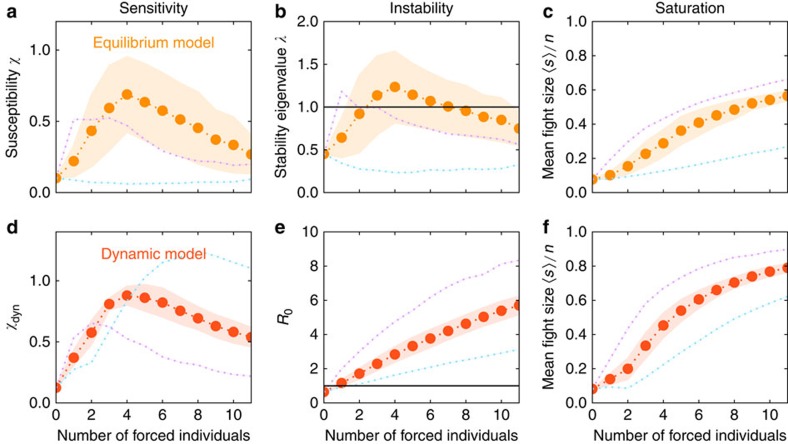
Simulation of forced individuals provides a biologically meaningful measure of distance from criticality. In equilibrium (**a**–**c**) and dynamic (**d**–**f**) models, forcing a few individuals to become simultaneously aggressive leads to a more sensitive system. (**a**,**d**) In each model, peak sensitivity is reached when on average 3–5 individuals are forced. Shaded regions indicate the standard deviation around the mean over different realizations of the chosen individuals. Purple and blue dotted lines indicate choices of forced individuals that maximize and minimize the resulting mean fight size. (**b**,**e**) The lowest-order stability measured in each model, corresponding to the eigenvalue *λ*_0_ in the equilibrium model and *R*_0_ in the dynamical model (Supplementary Note 3). A value above 1 in each case indicates instability to linear order. (**c**,**f**) The mean fight size. See Supplementary Note 4 for details about the ordering of forced individuals.

As shown in Fig. 3, both models predict a substantial increase in sensitivity when a few individuals are forced to be continuously involved in conflict. The system begins to saturate in sensitivity after 3–5 individuals are simultaneously forced. This result is similar to the finding that bird flocks require only a small proportion of informed individuals to correctly choose direction^18^,^19^. We use perturbation theory to demonstrate that this sensitivity arises from instability in each model (Supplementary Note 3, details on collective instability), a conclusion that is robust to uncertainties in model parameter estimates (Supplementary Note 1).

We find for both models that individuals vary in their sensitivity scores such that forcing participation of individuals with the top two to three sensitivity scores is sufficient to move the system close to a critical point (purple lines in Fig. 3).

## Discussion

Social systems must learn to tune the benefits of flexibility with the need for robustness. This can be achieved through collective forms of interaction that in effect tune the distance of a system from a critical point.

We are able to quantify through simulation the distance of a population from a finite critical point. The number of individuals (or, in principle, subgroups) that must be forced to reach peak sensitivity provides an operational definition of DFC and has the advantage of allowing the measurement of DFC in units that are natural to the system and hence mechanistically meaningful.

We find three to five individuals are sufficient, with significant individual variation in sensitivity scores. In both the equilibrium and branching process models, individuals with higher sensitivity scores are those who (by definition) exert greater influence on how far a system sits from the critical point. We find that individuals inducing the largest increase in average fight sizes correspond to those who promote the largest increase in sensitivity, as might be expected in any system with largely excitatory interactions.

The question of whether biological systems can control how responsive they are to environmental change is an ongoing debate in the criticality community (for example, refs 1, 20) and the closely related evolvability community (for example, refs 21, 22, 23, 24).

The sensitivity scores generated by each model are capturing different tuning mechanisms. In the case of the branching process model, we capture the spread of fight joining through direct interactions: one individual through its behaviour triggers the involvement of a second individual. Hence this model only allows that the target individuals, through their own fight-joining decisions, can up or down regulate their fight involvement. When a high sensitivity individual up or down regulates its own behaviour moving the system respectively closer to or further from the critical point, we call this direct tuning.

The equilibrium model, on the other hand, is agnostic to cause, recording any pairwise correlation in fight joining regardless of which individual triggered the joining event. As such it captures the full space of behavioural mechanisms leading to fight-joining: individuals can become involved in fights by up regulating their own fight involvement and also through changes to third-party behaviour. For example, policing (by third parties to the fight, see ‘Operational definitions' in Methods^25^) and other conflict management mechanisms reduce the frequency of redirected and directed aggression in the system and, in particular, towards the high sensitivity individuals, and hence dampen the fight-joining behaviour of these individuals. When a third party, like a policer, up or down regulates the behaviour of high sensitivity individuals we call this indirect tuning.

A natural question raised by the discovery that DFC can be tuned and that there are behavioural mechanisms in place that allow for this tuning is whether to favour robustness (increase DFC) or criticality (decrease DFC). This decision depends on three factors: (1) the adaptive utility of criticality—when does it make sense to sit near the critical point?, (2) the true state of the environment—is it uncertain or stable and predictable? and (3) the perceived state of the environment—the accuracy with which system components can detect and encode the environmental state.

The consequences of being near or at the critical point is that information can propagate quickly or more completely, with small changes to component behaviour inducing large-scale changes in both structure and function. Hence criticality allows the system to more easily reconfigure. We propose two hypotheses for tuning.

The first hypothesis we call the unspecified reconfiguration or evolvability hypothesis. The idea is that if the environment becomes uncertain after a period of stability, strategies adapted to the previously stable environmental state will be ill-suited to both the increasing uncertainty as well as to any new state the environment settles into. Hence the system moves toward the critical point to allow a reconfiguration to take place. To make this more concrete consider the model system studied here. Moving towards criticality changes the distribution of fight sizes such that there are more large fights. Social reconfiguration becomes more likely with large fights because large fights cost more^13^ and costly conflict can lead to changes in alliances, coalitions and the power structure, which controls the cost of conflict management^26^,^27^. Hence the opportunity for large-scale reorganization of social structure becomes possible, though it is not possible to predict the specific form the social structure will take. We predict that unspecified reconfiguration is adaptive when the environment is uncertain. Hence when the state of the environment is stable or very slowly changing (represented, for example, by a delta function), we expect the optimal choice is to increase DFC. When the state of the environment is uncertain, the system should decrease DFC. Unspecified reconfiguration can be thought of as an evolvability mechanism.

The second hypothesis we call directed reconfiguration. The idea here is that if a change occurs in the environment and this change has been observed before (for example, a predator appears), it may make sense to quickly switch from the current social configuration (say foraging) to one more appropriate for the perturbation^7^,^28^. Decreasing DFC allows information about the perturbation to quickly spread across the system so that components can rapidly exhibit behaviour appropriate for the new, but ‘understood,' environmental state. Directed reconfiguration is a mechanism for switching between behavioural states at the aggregate level as described in refs 7, 10.

For tuning to be adaptive, the state of the environment must also be correctly perceived by the tuning agent(s). To make this clear, consider the following: a flock of birds in search of food with high and low sensitivity individuals. Some individuals in the flock have poor eyesight and so misjudge the location of food and others have good eyesight. If the high-sensitivity birds (those whose position in the network means changes to their behaviour can be felt globally) are also those with poor eyesight, the system may be inappropriately driven away from food sources. If on the other hand the good eyesight birds are also highly sensitive, the system will be appropriately driven towards food. In a similar way, tuning DFC will require accurate perception of the most beneficial change. Hence in order for DFC to be tunable and for that tuning to be adaptive in a biological system these two types of heterogeneity must be aligned: high sensitivity score individuals must also be good detectors of environmental state. Neither of these types of heterogeneity has received much attention in the collective behaviour, criticality, or biological phase transition literatures. Consequently little is known about how common it is for these types of heterogeneity to be aligned or whether there are in some systems mechanisms to bring them into alignment.

In our model system, both types of heterogeneity appear to be aligned. Results of earlier work^29^ suggest the individuals with the highest sensitivity scores also are those who serve as good detectors of environmental state, with environmental state in this case corresponding to whether the underlying ‘social order' is stable or uncertain. The idea is as follows: some individuals register stressful periods more visibly (in their ‘health state') than the other group members due to their social position^30^. In our study system, these individuals are those perceived least capable of winning fights and who consequently sit in the lowest 10% of the social power distribution—these individuals pay a relatively high cost (in terms of aggression received) for social interactions^25^,^31^. ‘Health state', which fluctuates over days and weeks, is a slow variable^29^,^32^, compared with behaviours like aggression and submission which fluctuate on an hourly or daily scale. When these low-power individuals are healthy, the system can be said to be in a stable, low variance period. When these individuals are stressed above some baseline level, social dynamics are becoming uncertain. In our study system, the high sensitivity score individuals tend to be these weak individuals. Other individuals can adjust their propensity to attack or protect (for example, through policing^26^) these weak individuals based on their perceived health state and, in doing so, efficiently adjust DFC.

All biological systems need to balance stability and robustness with the need for rapid adaptive change (for example, refs 22, 33, 34). Yet many biological systems are observed to sit near a critical point^1^, which suggests a lack of robustness. This apparent conflict can be resolved by the discovery that DFC can be tuned (or computed) through realistic biological mechanisms. Tuning DFC allows for switching between stability and criticality, providing a means for accessing alternative social structures that may be more appropriate if and when the environment should change^29^,^32^.

This discovery raises many new questions: it is one thing for tuning to be possible and another to tune adaptively. We propose robustness is a good strategy when the environment is stable and low variance. Criticality is a good strategy when the environment is uncertain. In addition, in order for tuning to be adaptive the state of the environment must be accurately perceived by, or mirrored in, the individuals whose behaviour changes the DFC. This appears to be the case in our system but may present a crucial evolutionary challenge for other systems. Future comparative studies are required to quantify the range of DFC across social groups within a species, as well as across biological systems more generally, and to study how DFC might be controlled in other systems. We can then assess whether variation in DFC and its control are correlated with the rate of change in the environment and or environmental uncertainty.

## Methods

### Ethics statement

The data collection protocol was approved by the Emory University Institutional Animal Care and Use Committee and all data were collected in accordance with its guidelines for the ethical treatment of nonhuman study subjects.

### Study system

The data were collected by JCF in 1998 from a large group of captive pigtailed macaques (*Macaca nemestrina*) socially housed at the Yerkes National Primate Center in Lawrenceville, Georgia. Pigtailed macaques are indigenous to south East Asia and live in multi-male, multi-female societies characterized by female matrilines and male group transfer upon onset of puberty^35^. Pigtailed macaques breed all year. Females develop swellings when in Œstrus. Macaque societies more generally are characterized by social learning at the individual level, social structures that arise from nonlinear processes and feed back to influence individual behaviour, frequent non-kin interactions and multiplayer conflict interactions, the cost and benefits of which can be quantified at the individual and social network levels^25^,^26^,^31^,^36^,^37^,^38^.

The study group contained *n*=48 socially mature individuals (we exclude non-mature individuals because their behavioural strategies are still developing and so are non-stationary over short timescales) and 84 individuals in total. Socially mature males were at least 48 months and socially mature females were at least 36 months by study start. These thresholds correspond to approximate onset of social maturity in pigtailed macaques. The study group had a demographic structure approximating wild populations and subadult and adult males were regularly removed to mimic emigration occurring in wild populations. All individuals, except 8 (4 males, 4 females), were either natal to the group or had been in the group since formation. The group was housed in an indoor–outdoor facility, the outdoor compound of which was 125 × 65 ft.

Pigtailed macaques have frequent conflict and employ targeted intervention and repair strategies for managing conflict^25^. Data on social dynamics and conflict were collected from this group over a stable, four month period. Operational definitions are provided below.

### Operational definitions

#### Fight

It includes any interaction in which one individual threatens or aggresses a second individual. A conflict was considered terminated if no aggression or withdrawal response (fleeing, crouching, screaming, running away and submission signals) was exhibited by any of the conflict participants for 2 min from the last such event. A fight can involve multiple individuals. Third parties can become involved in pair-wise conflict through intervention or redirection, or when a family member of a conflict participant attacks a fourth-party. Fights in this data set ranged in size from 2 to 31 individuals, counting only the socially mature animals. Fights can be represented as small networks that grow and shrink as pair-wise and triadic interactions become active or terminate until there are no more individuals fighting under the above described two minute criterion. In addition to aggressors, a conflict can include individuals who show no aggression or submission (for example, third-parties who simply approach the conflict or show affiliative/submissive behaviour upon approaching, and recipients of aggression who show no response to aggression (typically, threats) by another individual). Because conflicts involve multiple actors, two or more individuals can participate in the same conflict but not interact directly.

In this study only information about fight composition (which individuals were involved) is used. Only fights that included two or more socially mature individuals were used in the analysis; the data includes *N*=994 such fights. We do not consider internal aspects of the fight, such as who does what to whom, except for the order of each individual's first involvement in the fight (used to estimate time-ordered conditional probabilities for use in the dynamical branching process model). Time data were collected on fight onset and termination but are not used in these analyses.

#### Power

The degree of consensus among individuals in the group about whether an individual is capable of using force successfully^26^,^27^. In previous works, we showed that consensus can be quantified by taking into account the total number of subordination signals an individual receives and multiplying this quantity by a measure of the diversity of signals received from its population of signalers (quantified by computing the Shannon entropy of the vector of signals received by individual *i*)^26^. In pigtailed macaque societies, the subordination signal is the silent bared teeth display^36^ emitted outside the conflict context during pass-byes and affiliative interactions. The distribution of power in our study group is heavy tailed, such that a few individuals are disproportionately powerful.

#### Policing

A policing intervention is an impartial intervention performed by a third party into an ongoing conflict^25^. Three males and one female perform the majority of effective policing interventions but only the three males (Eo, Qs, Fo) specialize on policing^12^. These four individuals occupy the top four spots in the power distribution^25^,^27^.

#### Redirection

A redirection occurs when an aggressor, recipient, or intervener directs aggression at a third (or fourth) individual who was not its original target or attacker. The target of the redirection may not have been involved in the fight until the redirection, or may have been involved in the fight but interacting with individuals other than the redirecting individual.

### Data collection protocol

The data were collected by a trained observer (J.C. Flack). The observer spent roughly 100 h before data collection learning to recognize individuals and accurately code their behaviour from the observation tower above the monkey compound. Accuracy was validated by a second trained observer (F.B.M. de Waal). J.C.F. also evaluated coding accuracy using video. Coding accuracy was nearly 100%.

During observations all individuals were confined to the outdoor portion of the compound and were visible to the observer. The ≈150 h of observations occurred for up to 8 h daily between 1,100 and 2,000 h over a 20-week period from June through October 1998, and were evenly distributed over the day. Conflict and signalling data were collected using all-occurrence sampling in which the entire conflict event is followed from start to finish and all participants and their behaviour are recorded.

Provisioning occurred before observations and once during observations at approximately the same time each day. The group was stable during the data collection period (defined as no reversals in status signalling interactions resulting in a change to an individual's power score; see ref. 26).

### Model descriptions and justification

We first evaluate which of three basic, empirically grounded fight-joining models explain our macroscopic observable, the distribution of fight sizes. We only accept a model if it is both consistent with the measured, microscopic data and can recover in simulation the observed, measured macroscopic output. Hence all of our models are closely tethered to the measured data and biological details of our model system.

It is important to realize that the parameters in the maximum entropy and branching models come from the microscopic data. The models do not assume prescribed values for parameters but are perhaps better viewed as hypotheses about the ways in which the measured, microscopic detail is connected to observed macroscopic patterns.

All models assume that events external to the system do not create correlations in behaviour. The data were collected in a controlled, captive setting designed to minimize the influence of external events, and we have no evidence for important, consistent external forcers of conflict.

The independent model assumes individuals do not respond to each other but instead join fights without regard to who else is fighting. In this case, perturbations to individual conflict behaviour would have no additional effect on group behaviour.

The independent model fails to recover both the observed distribution of fight sizes (Fig. 1) and the observed significant pairwise correlations (Supplementary Fig. 7).

Individuals sometimes randomly join fights and sometimes the decision to fight reflects strategic interactions at a pairwise level^13^. We capture this interpretation of the microscopic data using a maximum entropy approach, which corresponds to the spin-glass model of magnetic systems in physics. Because the model is parameterized by the data, it is empirically grounded and serves as a valid biological hypothesis. However we note that it is less mechanistically specific than the branching process described below: spin-glass interactions are not directional or time-ordered, but rather operate symmetrically and over the timescale of an entire fight bout.

The pairwise maximum entropy model, with parameters fit from the microscopic data, recovers well the distribution of fight sizes (Fig. 1). The good performance of the model leads to the prediction that the sensitivity *χ* is about twice that of a system with the same conflict frequencies but no strategic interactions.

Another reasonable interpretation of the microscopic data is that the random component of fight-joining decisions is very small and the decision to fight reflects strategic interactions at a pairwise level. This leads to a branching process model that, with parameters fit from the microscopic data, also recovers the observed distribution of fight sizes. The collective behaviour produced by this model can be simply understood in terms of a single parameter, the branching ratio. Additionally, branching process models like this one have been used in previous work to explore other aspects of conflict dynamics in this system, including the role of policing and other forms of third-party intervention in the infectivity of aggression^12^.

### Independent model inference

The independent model consists of individuals participating in conflict randomly, with the frequencies of individual appearance equal to their empirically measured, heterogeneous values *f*_*i*_=〈*x*_*i*_〉. Naively, this can be written as a relative negative log likelihood 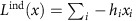, and is equivalent to a maximum entropy model that matches only the frequencies *f*_*i*_. (The relative negative log likelihood *L*(*x*) of state *x* is related to the likelihood *p*(*x*) by *p*(*x*)=exp(−*L*(*x*))/*Z*, where *Z* is a normalization constant set by the constraint that the sum of likelihoods over all states is one. In statistical physics, *Z* is the partition function and *L*(*x*) is proportional to the free energy of state *x*.)

However, as detailed in ‘Operational definitions' in the Methods section, a fight was operationalized for these analyses as involving two or more socially mature individuals. (Observed fights that involved only juveniles and 0 or 1 mature individuals are therefore excluded.) Correspondingly, we forbid our models from producing fights of size smaller than two. We treat this as an additional constraint on the model. The resulting maximum entropy model is then the one in which the likelihood of states with fewer than two individuals present is taken to zero. This corresponds to a relative negative log likelihood


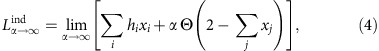


where Θ(*z*) is 0 when *z*≤0 and 1 when *z*>0.

In the unconstrained case (*α*=0), we can easily solve for *h*_*i*_:


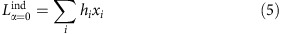



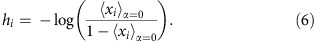


We must now solve numerically for *h*_*i*_ to match the empirically measured *f*_*i*_=〈*x*_*i*_〉=〈*x*_*i*_〉_*α*→∞_. To accomplish this, note that the unconstrained model will have modified statistics:


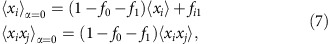


where *f*_0_ is the frequency of size zero fights, *f*_*i*1_ is the frequency of size one fights consisting solely of individual *i*, and 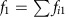 is the overall frequency of size one fights (all measured in the unconstrained model). In terms of unconstrained individual frequencies, these are


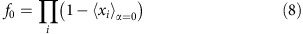



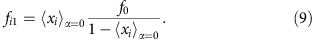


We use an iterative procedure to solve equation (7) for 〈*x*_*i*_〉_*α*_=_0_, which are then used in equation (6) to find the fields. Finally, samples from the independent model (equation (4)) are produced by sampling using equation (5) and simply discarding samples in which fewer than two individuals appear. For our data, this results in discarding about 17% of samples produced with equation (5).

### Pairwise maximum entropy model inference

We next constrain our equilibrium maximum entropy model to match the frequencies of appearance of both individuals and pairs of individuals. This model is known to be the spin-glass Ising model^39^, with relative negative log likelihood


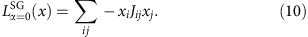


As in the independent model, the likelihood of fights with fewer than two individuals is taken to zero:


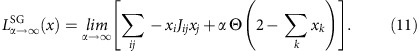


The statistics we fit in the equilibrium model are the individual and pairwise frequencies of appearance:


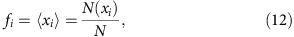



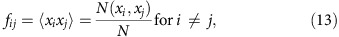


with *N*(*x*_*i*_) and *N*(*x*_*i*_, *x*_*j*_) representing, respectively, the observed number of appearances in unique fights of the individual *i* and the pair *i*, *j*.

We would like to fit the individual and pairwise frequencies of appearance to the precision that the data supports. As a measure of the goodness of fit, we use the average of normalized residuals


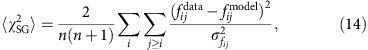


where


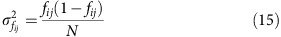


is the expected variance of each residual due to finite *N*, and repeated indices are understood as individual frequencies: *f*_*ii*_=*f*_*i*_. A good fit is expected to have 〈*χ*^2^〉≈1.

To perform fitting, we use a simplified method that starts with the mean-field solution and varies a single parameter corresponding to weighting a non-interacting prior. Specifically, we make use of the *L*_2_-regularized mean-field entropy of ref. 40. The regularization consists of a Gaussian prior with a form designed to make the mean-field case easily solvable, corresponding to an additional term in the relative negative log-likelihood


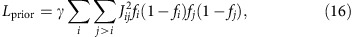


where *γ* is the strength of the prior, which favors interactions *J*_*ij*_ that are smaller in magnitude. The mean-field solution under this regularization is ref. 40


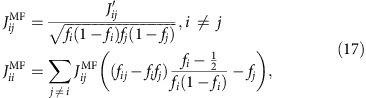


where *J*′ is the matrix that has the same eigenvectors *v*_*q*_ as the correlation matrix


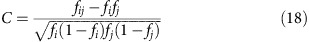


and eigenvalues 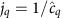, where 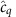 are regularized versions of *C*'s eigenvalues *c*_*q*_:


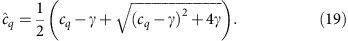


This regularization is typically used in a Bayesian sense to avoid overfitting, where a typical value of the regularization strength is *γ*=1/(10*N f*^2^(1–*f*)^2^), with *N* the number of samples and *f*=*n*^−1^∑_*i*_ *f*_*i*_ the average individual frequency^40^. Avoiding overfitting, however, still typically requires either enormous *N* (for example, ref. 39) or restricting the effective number of fit parameters via an expansion (for example, ref. 40). In our case, *N* is fundamentally limited in that we are describing a stable social epoch of finite duration. In addition, typical high-temperature expansions cannot easily incorporate the restriction that fights have a minimum number of participants (the *α* term in equation (11)).

Alternatively one can treat *γ* as a fitting parameter that interpolates between the mean-field solution (which we find overestimates the strength of interactions) and the case of independent individuals. Although it is not *a priori* obvious that varying this single parameter will be enough to fit the observed statistics within expected statistical fluctuations, we find that this is true for our data. Numerically sampling from the distribution defined by equation (11) with the regularized mean-field *J* from equation (17), we minimize 〈*χ*^2^〉 from equation (14) as a function of *γ*. Sampling is performed using a standard Metropolis Markov Chain Monte Carlo approach. In evaluating the fit, we choose the number of samples to scale with the number of data samples, using *N*_samples_=20*N*.

As a simple check that this inference approach is not biased to find more sensitive systems, we infer a pairwise model using data produced by the independent model and compare its susceptibility to the known exact value (equation (11) in Supplementary Note 3) in Supplementary Fig. 1. The resulting inferred model has susceptibility that stays close to the true value for small *h*_ext_ and remains smaller than the true value for larger *h*_ext_.

The variation in inferred *J*_*ij*_ parameters over individuals can be seen in the lefthand plot of Supplementary Fig. 3. Interaction terms are slightly more often positive than negative, corresponding to excitatory interactions.

### Branching process inference

In the branching process model, we use time-ordered appearance data, fitting the time-ordered conditional appearance probabilities


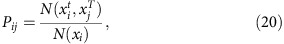


where 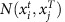 counts the number of times individual *j* appears in the same fight bout as individual *i*, but at a later time *T*>*t*. Note that this is different than previous work in inductive game theory^13^: there (time directed) correlations were measured between individual appearances in separate fight bouts, whereas here we measure correlations within fight bouts.

The parameters we vary in the heterogeneous branching process model are the single-step conditional probabilities *p*_*ij*_, which measure the probability that individual *j* appears in step *t*+1 of the branching process given that individual *i* appeared in step *t*. Being probabilities, *p*_*ij*_ are constrained to values 0≤*p*_*ij*_≤1. (Note that the branching model is thus limited in the extent to which it can represent inhibitory interactions, for example, if *i*'s involvement in the fight deters *j* from joining.) We modify this constrained optimization problem into an unconstrained one by defining 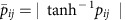 and performing the optimization over the (unconstrained) 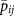 parameters.

In the branching process simulation, the first individual to join each fight bout is chosen randomly with probability proportional to the frequency with which each individual appears at the beginning of fights in the data. At each subsequent time step in the branching process, each individual *j* who has not yet been activated in the current fight has a probability of joining equal to the sum of *p*_*ij*_ for all *i* active in the previous time step. The fight bout concludes when no individuals are active in a given time step. As discussed above, fight bouts that do not grow beyond a single individual are discarded.

Analogously to the case of the equilibrium maximum entropy model, the branching process parameters are fit by minimizing


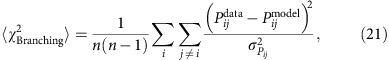


where


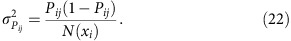


We use a standard Levenberg–Marquardt algorithm (scipy.optimize.leastsq) that uses individual residuals and a lowest-order approximation for the Jacobian with respect to parameters. We find that restarting the Levenberg–Marquardt routine every 10 steps (effectively resetting its damping parameter to avoid unnecessarily small steps arising from its assumption of a non-stochastic objective function) produces faster convergence with fewer samples required for each estimate of the residuals and Jacobian. Minimization is stopped once 〈*χ*^2^〉 defined in equation (21) falls below 1. In addition, we find that switching between simple gradient descent steps and Levenberg–Marquardt minimizations allows for more efficient fitting of larger *P*_*ij*_.

The resulting inferred redirection probabilities *p*_*ij*_ are visualized in Supplementary Fig. 2 and the righthand plot of Supplementary Fig. 3.

### Data availability

The data that support the findings of this study are available from J.C.F. upon reasonable request.

## Additional information

**How to cite this article:** Daniels, B. C. *et al*. Control of finite critical behaviour in a small-scale social system. *Nat. Commun.* **8,** 14301 doi: 10.1038/ncomms14301 (2017).

**Publisher's note:** Springer Nature remains neutral with regard to jurisdictional claims in published maps and institutional affiliations.

## Supplementary Material

Supplementary Information Supplementary figures, supplementary table, supplementary notes and supplementary references.
