# Supplementary material for: Control of finite critical behaviour in a small-scale social system
Source: Nat Commun. 2017 Feb 10;8:14301. doi: 10.1038/ncomms14301 (PMC5309824; doi:10.1038/ncomms14301)
Supplement: Supplementary Information — Supplementary figures, supplementary table, supplementary notes and supplementary references. [file ncomms14301-s1.pdf]

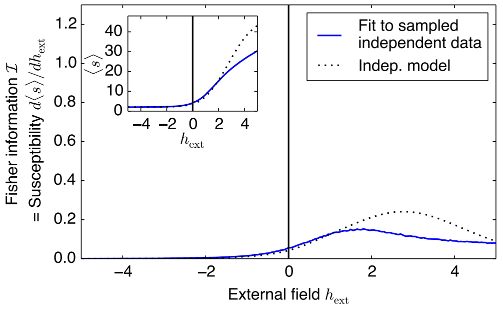

Supplementary Figure 1. Performing the second-order maximum entropy fitting procedure on an amount of data equal to the observed fights drawn from a noninteracting model produces a flat susceptibility curve comparable to the known exact susceptibility for this case.

Supplementary Note 1. PARAMETER UNCERTAINTY

The tightness with which we can constrain parameters in each of the models is limited by the finite amount of data, and this leads to uncertainty in the measurements of sensitivity, stability, and distance from criticality. The high dimensionality and nonlinearity of the mapping from model parameters to model predictions rules out direct analytical calculation of posterior distributions. Yet we can use three methods to approximate uncertainties and check that they do not qualitatively affect our results.

First, in the pairwise maximum entropy model, an asymptotic approximation to parameter uncertainties can be computed semi-analytically. Second derivatives of the log-likelihood with respect to parameters J_{ij} (the Fisher Information matrix) can be computed by estimating fourth-order statistics [1]:

$$\mathcal{I}_{\alpha\beta} = N\left(\langle x_\alpha x_\beta\rangle - \langle x_\alpha\rangle\langle x_\beta\rangle\right), \tag{1}$$

where N is the number of observations, and α and β refer to individuals or pairs (e.g., the entry of $\mathcal{I}$ at $\alpha = (1,2)$ and $\beta = 3$, corresponding to parameters J_{12} and J_{33}, is $N(\langle x_1 x_2 x_3\rangle - \langle x_1 x_2\rangle\langle x_3\rangle))$. The inverse of $\mathcal{I}$ produces the quadratic form describing fluctuations in inferred parameters when N is large. In particular, the lowest-order uncertainty in the value of a function g (such as the susceptibility or stability eigenvalue), can be written in terms of the gradient of g with respect to parameters J_{ij} and the eigenvalues F_α and corresponding eigenvectors $\vec{f_\alpha}$ of $\mathcal{I}$:

$$\sigma_g^2 = \sum_\alpha \left(\vec{\nabla} g \cdot \vec{f_\alpha}\right)^2 F_\alpha^{-1}. \tag{2}$$

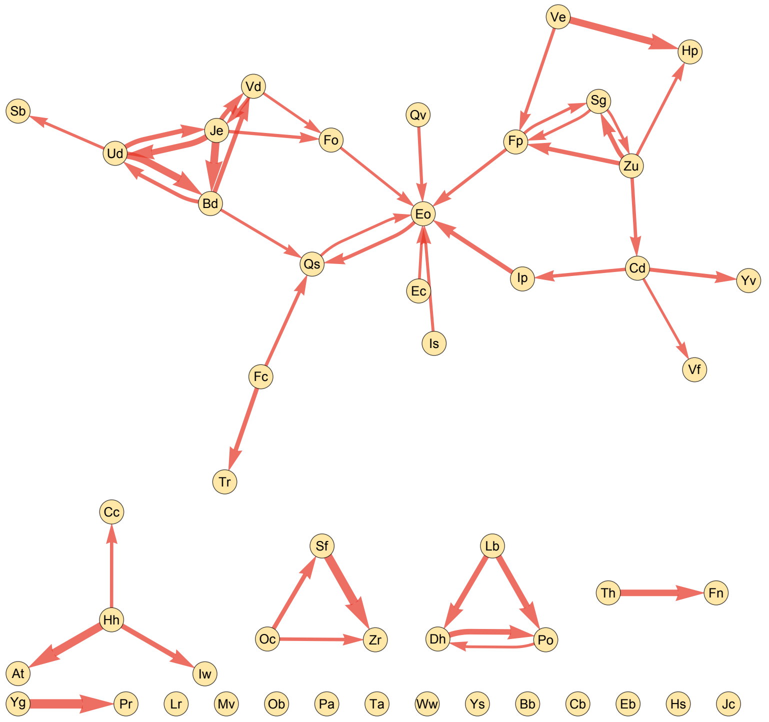

Supplementary Figure 2. A visualization of the inferred branching process graph. In the full branching process model, all possible directed pairwise interactions are included, but here we visualize the most important interactions by displaying those with triggering probabilities > 0.09. The thickness of each arrow corresponds to the probability of triggering, with the thickest lines correspond to a probability of about 1/3: for instance, when Yg appears, Pr will be triggered by Yg to join with probability of about 1/3.

Calculating the gradients of susceptibility χ and mean fight size s is somewhat messy but straightforward. The gradient of the stability eigenvalue λ, an eigenvalue of the non-symmetric matrix M, is

$$\vec{\nabla}\lambda = \frac{\vec{m}_L^T \cdot \vec{\nabla} M \cdot \vec{m}_R}{\vec{m}_L^T \cdot \vec{m}_R}, \tag{3}$$

where $\vec{m}_L$ and $\vec{m}_R$ are the left and right eigenvectors of M corresponding to λ. This analytical method produces uncertainty estimates shown in the top row of Supplementary Figure 4.

Second, we can simultaneously estimate uncertainties from finite sampling and check for consistent inference using a bootstrapping method, (1) sampling from each model a number of fights equal to the number of observed fights and (2) running the inference procedure

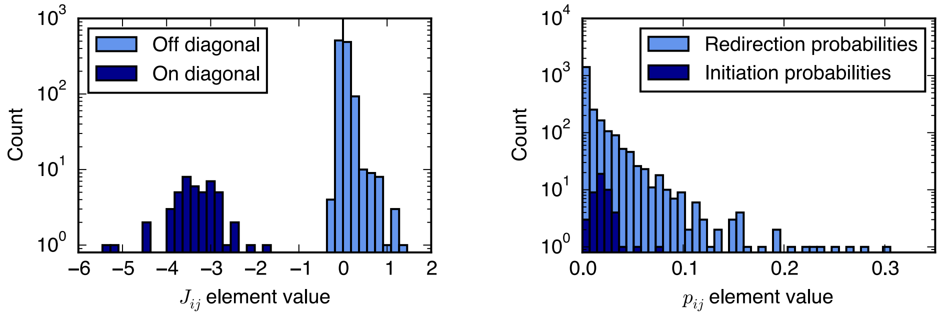

Supplementary Figure 3. Histograms of inferred parameter values in each model. (Left) Parameters J_{ij} of the maximum entropy pairwise model (with only one of each symmetric pair counted for the off-diagonal case). (Right) Conditional redirection probabilities p_{ij} of the branching process model (light blue) and the probabilities of each individual being the first to join each fight (dark blue).

on the sampled data. Variance in the results over multiple samplings is a straightforward estimate of the variance due to parameter uncertainty. The standard deviations over 10 samplings are shown in the middle row of Supplementary Figure 4 and for both the equilibrium and dynamic models in Supplementary Figure 5.

Third, a simple check for robustness of the results is to run the calculation on subsets of the data. In the bottom row of Supplementary Figure 4, we display results computed on two mutually exclusive halves of the data (corresponding to the in- and out-of-sample data in Supplementary Note 2).

All three methods confirm that our main qualitative findings, the peak in sensitivity and the system becoming unstable at positive h_{ext}, are not washed out by uncertainty in parameters.

Supplementary Note 2. MODEL EVALUATION

To check the performance of each of our models, we first compare statistics computed with the model to those computed on out-of-sample data. The results for a single choice of in-sample data are shown in Supplementary Figure 6. Half of the fights are randomly chosen as in-sample data, with the remaining treated as out-of-sample data to be predicted. We see that the independent model does not capture second- or third-order statistics nor the distribution of fight sizes, while both the equilibrium and dynamic models capture these

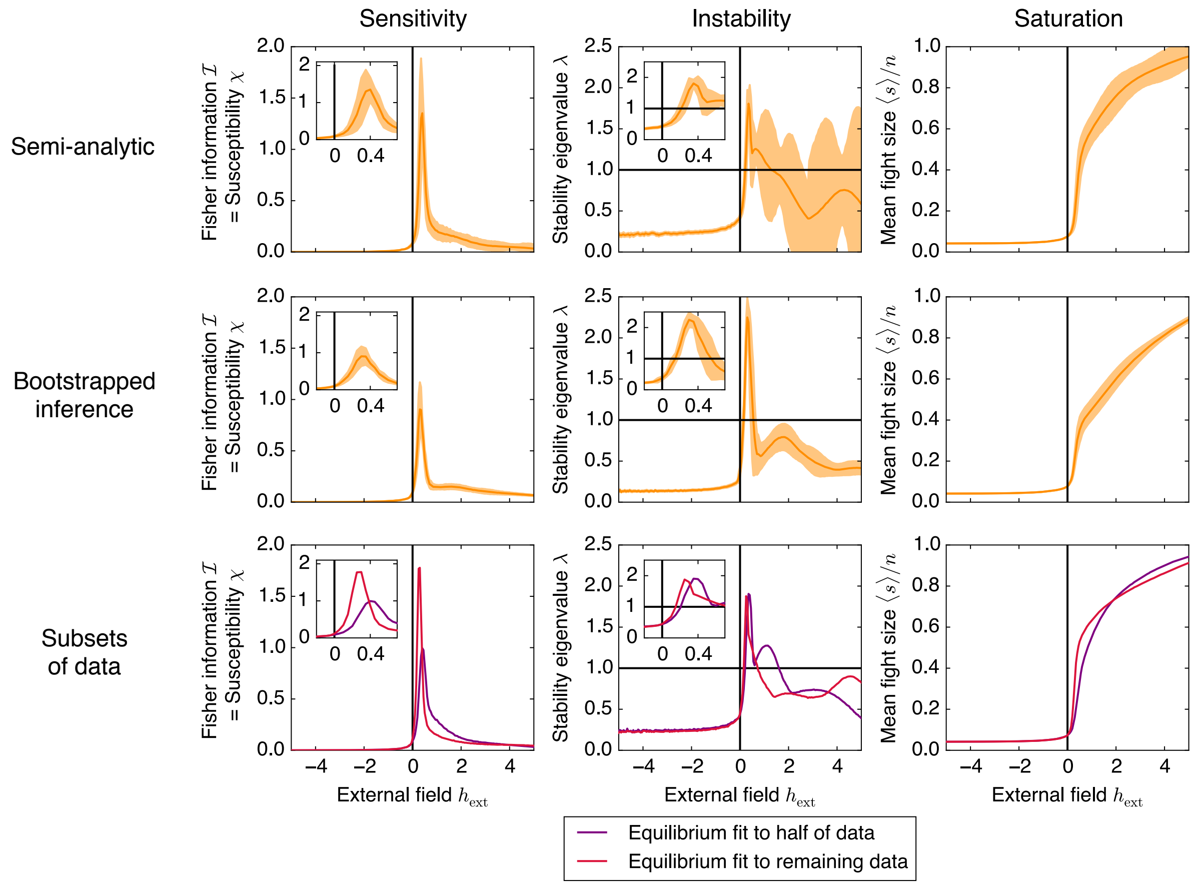

Supplementary Figure 4. Uncertainties in sensitivity, instability, and saturation, estimated using three methods. Insets zoom in on the peak in sensitivity and instability, which remains unambiguous in all cases. (Top row) A semi-analytic approximation of uncertainties, with shaded areas representing $\pm\sigma$ as calculated using Eq. (2), and means calculated from inference on the full dataset (as in Fig. 2). (Middle row) Means and standard deviations of results from the inference procedure applied to 10 sets of sampled data from the original fit model. (Bottom row) Results from inference applied to two distinct random subsets of the data.

to produce predictions that are roughly as accurate as using out-of-sample data.

Second, we can check that residuals lie within the bounds of expected statistical fluctuations from finite sampling. Shown in Supplementary Table 1, the equilibrium and dynamic models have squared residuals that are below but near the expected value $\langle \chi \rangle^2 = 1$, whereas the independent model is inadequate to describe the statistics. This is visualized in more detail with the distribution of residuals in Supplementary Figure 7.

We find no evidence of significant higher order correlations in the data (Supplementary

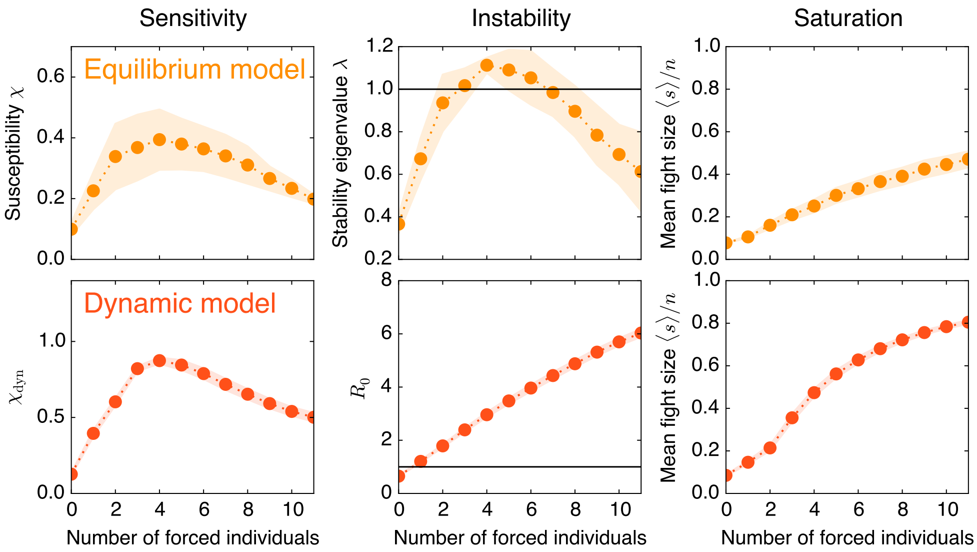

Supplementary Figure 5. Checking robustness of results to sampling from and re-inferring models. Plotted are means and standard deviations of results over 10 bootstrap inferences (with results first averaged over orderings of added individuals). Compare to Fig. 3.

Supplementary Table 1. Goodness of fit to data for the three models, calculated using Eq. (14) in the main text for the independent and pairwise maximum entropy models and Eq. (21) in the main text for the dynamic branching model. With $\langle \chi^2 \rangle \sim 1$, the equilibrium pairwise and dynamic branching models fit the data roughly within the precision afforded by the data. Overfitting, which would be indicated by $\langle \chi^2 \rangle \ll 1$, is avoided by using constrained minimization in the case of the spin-glass model (see Pairwise maximum entropy model inference in Methods) and by ending minimization once $\langle \chi^2 \rangle \leq 1$ in the case of the branching model (see Branching process inference in Methods).

	Independent model	*Equilibrium pairwise model*	*Dynamic branching model*
Random half of data	$\langle \chi^2 \rangle = 1.134$	$\langle \chi^2 \rangle = 0.459$	$\langle \chi^2 \rangle = 0.414$
All data	$\langle \chi^2 \rangle = 1.822$	$\langle \chi^2 \rangle = 0.597$	$\langle \chi^2 \rangle = 0.462$

Figure 7) and we therefore do not explore models with interactions of higher order. We note however that the resolution of higher order correlations is limited by the finite number of observed fights and relatively small frequency of individual participation. This cannot easily be remedied by collecting more data as the system is not at equilibrium over longer

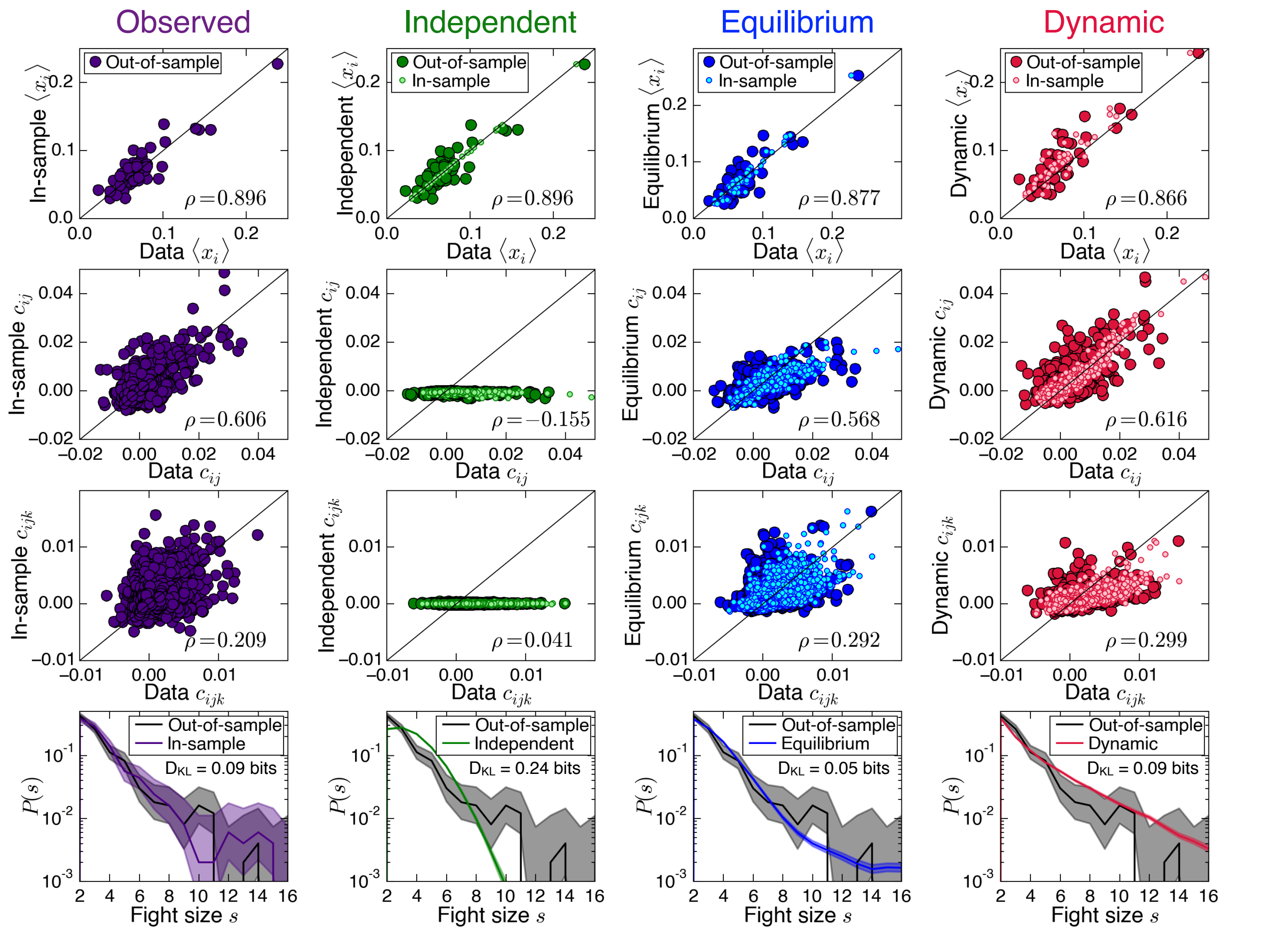

Supplementary Figure 6. The degree of fit for the noninteracting model (green), maximum entropy pairwise model (blue), and branching process model (red) to out-of-sample data, compared to the same for the in-sample data (indigo) to which the models are fit. For each model, 10^5 samples were taken to evaluate predicted statistics. Also shown on each plot is the Pearson correlation ρ between predicted and out-of-sample statistics (for individual, pairwise, and triplet statistics) or the Kullback-Leibler divergence D_{KL} between predicted and out-of-sample distributions (for fight sizes). (To avoid problems with large fight sizes that are never observed, D_{KL} is calculated only using fights of size ≤ 12.)

timescales. To deal with this, we must restrict the data we use in the analyses to collection windows defined by "socially stable periods" (see Methods Data collection protocol).

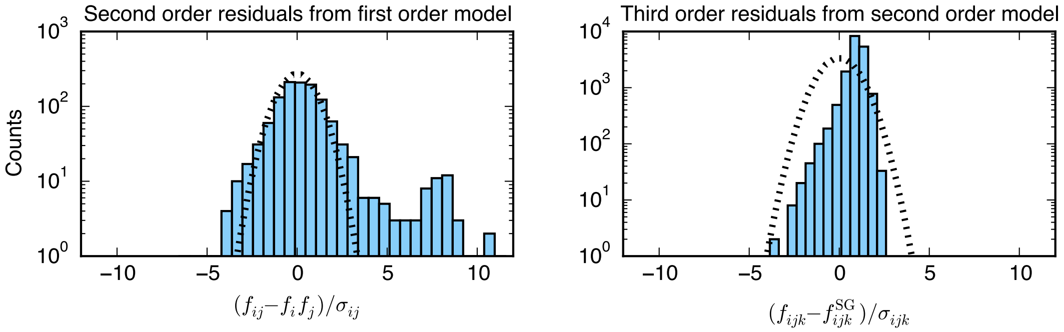

Supplementary Figure 7. Second- and third-order statistics in the conflict data. Second-order statistics (left) clearly violate the null expectation for a first-order independent model (dotted line), while third-order statistics (right) lie within expected fluctuations from a second-order model (dotted line). f_{ijk} is the empirical frequency with which each triplet appears in fights, f_{ijk}^{SG} is this frequency in the pairwise equilibrium model, and $\sigma_{ijk} = \sqrt{f_{ijk}(1 - f_{ijk})/N}$ is the expected standard deviation.

Supplementary Note 3. EVALUATING SENSITIVITY AND STABILITY

Phase transitions are typically identified as conditions under which the varying of a control parameter causes large-scale changes in the behavior of a system, in a way that sensitivity per individual (measured by, *e.g.*, specific heat or susceptibility) grows arbitrarily large with growing system size. This becomes possible only when there is a collective instability, meaning that the effective size of the perturbation (that starts, say, with a single individual) does not shrink as it spreads through the system but stays of constant size or grows (potentially affecting all individuals). Thus in a finite system, the combination of a peak in sensitivity and collective instability can be used as an indicator of a phase-transition-like state.

Sensitivity as Fisher information

In our finite system the notion of diverging sensitivity is arguably more accurately described in terms of information theory. Even when the idea of a phase transition becomes fuzzy in a finite system, the Fisher information measures something adaptively important: the degree to which individual scale perturbations are visible at the global scale, or, equivalently, the connection between the behavior of any individual and the behavior of the whole

[2, 3].

Analytical results for sensitivity in the independent model

Here we show that the sensitivity (susceptibility) to increased aggression in the independent model can be efficiently solved numerically. This is used to make a comparison with the pairwise equilibrium model in Fig. 2.

First, in the more analytically straightforward case in which we allow fights of size zero and one ($\alpha = 0$; see Independent model inference in Methods), the average fight size and susceptibility are

$$\langle s \rangle_{\alpha=0} = \sum_i \left(1 + \exp(h_i - h_{\text{ext}})\right)^{-1} \tag{4}$$

$$\chi_0 = \frac{\partial \langle s \rangle_{\alpha=0}}{\partial h_{\text{ext}}} = \sum_i \text{sech}^2(h_i - h_{\text{ext}}). \tag{5}$$

The partition functions of the constrained and unconstrained models, defined such that

$$p(\vec{x})_{\alpha=0} = \exp[-L_{\alpha=0}(\vec{x})]/Z_0 \tag{6}$$

$$p(\vec{x})_{\alpha\to\infty} = \exp[-L_{\alpha\to\infty}(\vec{x})]/Z_\infty, \tag{7}$$

are given by

$$Z_0 = \prod_i (1 + \exp h_i) \tag{8}$$

$$Z_\infty = Z_0 - 1 - \sum_i \exp h_i. \tag{9}$$

In terms of these values, when fights of size zero and one are forbidden ($\alpha \to \infty$), the average fight size and susceptibility become

$$\langle s \rangle_{\alpha\to\infty} = \frac{Z_0}{Z_\infty} \langle s \rangle_{\alpha=0} - \frac{\sum_i \exp h_i}{Z_\infty} \tag{10}$$

$$\chi_\infty = \frac{\partial \langle s \rangle_{\alpha\to\infty}}{\partial h_{\text{ext}}} = \frac{Z_0}{Z_\infty} \frac{\partial \langle s \rangle_{\alpha=0}}{\partial h_{\text{ext}}} - \frac{\sum_i \exp h_i}{Z_\infty} + \frac{Z_0}{Z_\infty} \langle s \rangle^2_{\alpha=0} - \langle s \rangle^2_{\alpha\to\infty}. \tag{11}$$

Details on collective instability in the branching process model

In the branching process model, the instability of the peaceful state is measured by R_0, the largest eigenvalue of the redirection probability matrix p_{ij}. As the system size approaches

infinity, $R_0 = 1$ corresponds to a well-defined phase transition. The fact that this "local" amplification factor is also indicative of a global transition relies on the infinite limit: In a finite system, cascades will be shortened when they reach individuals that have already been activated, and maximal sensitivity will happen at some $R_0 > 1$ [4], as we see in Fig. 3. We thus think of R_0 as measuring a "local" or lowest-order stability.

We note that in the branching model, as opposed to the equilibrium model, increasing activation never decreases instability. This is because (1) interactions in the branching model are assumed to be exclusively excitatory, whereas this is not the case in the equilibrium model, and (2) the equilibrium instability corresponds to perturbing the equilibrium mean-field state, which can become saturated, whereas the dynamic instability corresponds to perturbing the peaceful state. This produces the difference in behavior of instability measures in the two models in Fig. 3.

Details on collective instability in the pairwise equilibrium model

In an infinite system, the pairwise equilibrium model also has a phase transition under the condition of local instability, with a corresponding diverging sensitivity. In this case, instability can be quantified using the mean-field solution, connecting with a high-temperature expansion of spin-glass models.

One way to think about the continuous phase transition in an infinite spin-glass model is that it is the point at which the high temperature mean-field solution becomes unstable. Mean-field solutions are characterized by frequencies $\vec{f}$ of individual appearance ($f_i = \langle x_i \rangle$) that satisfy the self-consistency equation [5]

$$f_i = F_i(\vec{f}) \equiv \left[1 + \exp\left(J_{ii} + 2 \sum_{j \neq i} J_{ij} f_j \right) \right]^{-1}. \quad (12)$$

Intuitively, individual i's frequency of fighting is determined by the mean "field" it feels as a result of others' fighting. The function F_i encodes how i reacts to its environment, translating the mean fighting frequencies i sees into its own mean frequency. When Eq. (12) holds for every individual using a single set of frequencies $\vec{f}$, this defines the mean field solution.

Now imagine perturbing fighting frequencies $\vec{f}$ by a small $\Delta\vec{f}$. This will typically no longer be a solution of Eq. (12). But if we repeatedly apply the function F to $\vec{f} + \Delta\vec{f}$, we

can imagine two possibilities: we might end up back at $\vec{f}$ (so that $\lim_{n\to\infty} F^n(\vec{f}+\Delta\vec{f}) = \vec{f}$), or we might get further and further from $\vec{f}$. We will call the first case a "stable" mean field solution and the second case "unstable".

For small perturbations $\Delta\vec{f}$, we can distinguish these two cases by taking a derivative to perform a linear stability analysis. Specifically,

$$F_i(\vec{f}+\Delta\vec{f}) \approx F_i(\vec{f}) + \sum_j \frac{\partial F_i}{\partial f_j}\Delta f_j, \tag{13}$$

and to converge back to $\vec{f}$ for every perturbation, we must have that the updated perturbation along each direction has shrunk. This corresponds to a condition on the eigenvalues λ_α of the derivative matrix $M_{ij} \equiv \partial F_i/\partial f_j$; the state is stable if

$$|\lambda_\alpha| < 1 \quad \forall\ \alpha. \tag{14}$$

Thus the eigenvalue λ with largest magnitude determines stability.

We can write this derivative matrix M more explicitly by taking the derivative of Eq. (12) and assuming that we are at the fixed point ($\vec{f} = F(\vec{f})$):

$$\begin{aligned} M_{ij} &= \frac{\partial F_i}{\partial f_j} \\ &= -2(1-\delta_{ij})J_{ij}\exp\left(h_i + 2\sum_{j\neq i} J_{ij} f_j\right) f_i^2 \\ &= -2(1-\delta_{ij})J_{ij}\frac{1-f_i}{f_i} f_i^2 \\ &= -2(1-\delta_{ij})J_{ij} f_i(1-f_i). \end{aligned} \tag{15}$$

Thus M is a matrix analogous to p_{ij} in the branching process model in that its spectrum is informative about how perturbations grow or shrink. Specifically, we use the magnitude λ of the largest eigenvalue of M as a measure of stability of the system. When $\lambda > 1$, we expect the system to be unstable to perturbation.

This condition on the stability of mean field theory can be shown to be equivalent to the condition that identifies the spin-glass transition in an infinite system. Specifically, instability of the high-temperature mean-field expansion happens only below the spin-glass temperature [6, 7]. To lowest order in $1/T$ (corresponding to lowest order in J_{ij} or $1/N$), the mean-field free energy has the form (following [8])

$$A = \sum_i f_i \log f_i + (1-f_i)\log(1-f_i) - \sum_i \sum_{j\neq i} J_{ij} f_i f_j - \sum_i J_{ii} f_i, \tag{16}$$

which when differentiated produces the self-consistency equation (12). Taking a second derivative,

$$\frac{\partial^2 A}{\partial f_i \partial f_j} = \delta_{ij} \frac{1}{f_i(1-f_i)} - (1-\delta_{ij}) 2 J_{ij} \equiv \Lambda_{ij}, \tag{17}$$

which defines stability to this order when all eigenvalues of Λ_{ij} are positive [7]. Because $f_i(1-f_i) > 0$, this condition is the same as all eigenvalues of $f_i(1-f_i)\Lambda_{ij} = \delta_{ij} - M_{ij}$ being positive (where M is defined in Eq. (15)), which is in turn equivalent to the above condition that all eigenvalues of M_{ij} are less than 1.

To create a homogeneous finite system poised at the transition defined by the eigenvalue λ (shown as the red dotted curve in Fig. 2), we define a homogeneous positive h and negative J that make each individual's frequency $f = 1/2$ and $\lambda = 1$.

Supplementary Note 4. ORDERING OF FORCED INDIVIDUALS

In Fig. 3, the magenta and blue lines demonstrate the potential heterogeneity of responses when different individuals are forced. The magenta lines demonstrate the effect of forcing individuals in an order that maximizes the resulting average fight size at each step, and blue in an order that minimizes it. The individuals are re-sorted each time another is forced as this can affect the order (for instance, forcing one individual in a strongly-correlated clique can decrease the effect of forcing other individuals in that clique). In Supplementary Figure 8, we contrast the case in which individuals are sorted by their effect on the original, unperturbed state of the system. The qualitative results are the same as in Fig. 3.

Supplementary Note 5. THERMODYNAMIC DERIVATIVES AND FISHER INFORMATION IN THE EQUILIBRIUM MODEL

Quite generally for equilibrium models, it can be shown that the Fisher information is deeply related to important thermodynamic derivatives. The Fisher information is defined as [9]

$$\mathcal{I}(\mu) = \int \left(\frac{\partial \log p(x)}{\partial \mu} \right)^2 p(x) \, \mathrm{d}x, \tag{18}$$

where μ parameterizes a distribution $p(x)$ describing the behavior of a system, and x represents any number of relevant measurable system state variables. Generalized to multiple parameters, the Fisher information matrix is a fundamental object in information geometry,

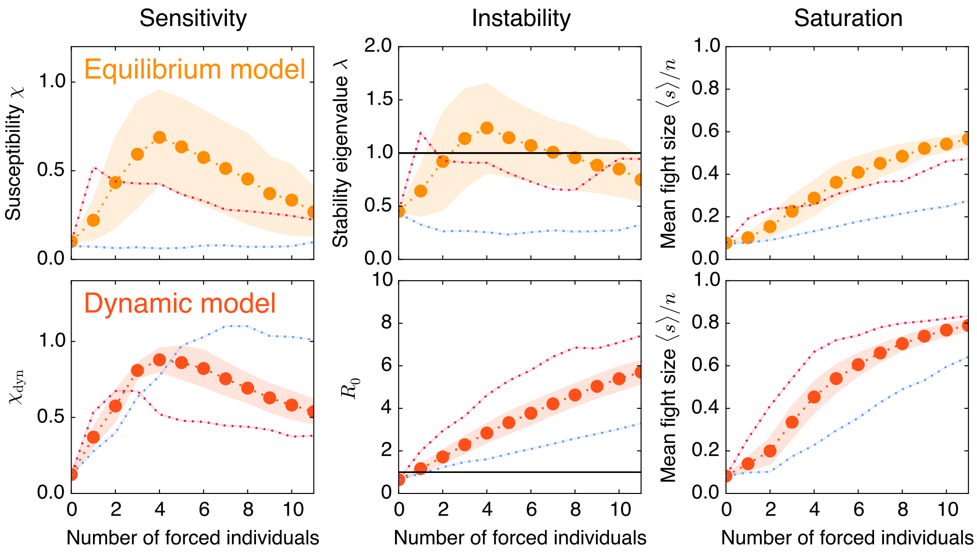

Supplementary Figure 8. Same as Fig. 3, except that the magenta and blue lines here show results when forced individuals are chosen as those with the largest and smallest effect on the mean fight size of remaining individuals when forced in the otherwise unperturbed system. (Fig. 3 reperforms this optimization after adding each individual.)

and forms a Riemannian metric that becomes singular precisely at phase transitions [3, 10]. $\mathcal{I}(\mu)$ is typically used to measure the amount of information about μ that can be inferred from draws from $p(x)$. Conversely, if we view individuals as controlling local parameters μ, $\mathcal{I}(\mu)$ measures the degree of control individuals have on group behavior. Then phase transitions, having diverging $\mathcal{I}$ as $N \to \infty$, correspond to individuals having arbitrarily large effects. But even at finite N, $\mathcal{I}$ measures the amplification of individual information to the global scale. In this sense, $\mathcal{I}$ becomes a straightforward, useful measure of the degree to which a system's behavior is "collective."

Another intuitive meaning comes in terms of the Kullback-Leibler divergence: $\mathcal{I}(\mu)$ represents how quickly the KL-divergence increases as μ is changed, such that

$$D_{\rm KL}\left(p(x|\mu)||p(x|\mu+\Delta\mu)\right) = \mathcal{I}(\mu)(\Delta\mu)^2/2 + O(\Delta\mu^4). \tag{19}$$

Thus the Fisher information measures how quickly the modified distribution becomes distinguishable from the original as μ is varied, and if logs are taken with base 2, $\mathcal{I}(\mu)$ has units of bits per [unit of μ]^2.

In the case of an equilibrium system described by a Boltzmann distribution, the Fisher

information with respect to a local field μ is particularly simple, equal to the derivative of the mean of its conjugate variable x_μ, the generalized susceptibility $\mathcal{I}(\mu) = \frac{\partial}{\partial\mu}\langle x_\mu \rangle$. This example provides a clear link between thermodynamics and information theory. (Yet the Fisher information measure is not limited to equilibrium models, generalizing to dynamic out-of-equilibrium systems by simply interpreting $p(x)$ in Eq. (18) as a distribution over relevant output measurements given some known initial conditions.)

This connection between Fisher information and thermodynamic derivatives is well-established [3]. Assume we have a system whose distribution over possible states x takes the form of a Boltzmann distribution:

$$p(x) = Z^{-1} e^{-L(x)}. \tag{20}$$

Taking a derivative of $\log p(x)$,

$$\frac{\partial \log p(x)}{\partial \mu} = -\frac{\partial L(x)}{\partial \mu} + Z^{-1} \sum_x \frac{\partial L(x)}{\partial \mu} e^{-L(x)} \tag{21}$$

$$= \left\langle \frac{\partial L}{\partial \mu} \right\rangle - \frac{\partial L(x)}{\partial \mu}, \tag{22}$$

which, when inserted in Eq. (18), gives

$$\mathcal{I}(\mu) = \left\langle \left(\frac{\partial L}{\partial \mu} \right)^2 \right\rangle - \left\langle \frac{\partial L}{\partial \mu} \right\rangle^2. \tag{23}$$

This shows that Fisher information is equal to the variance of the derivative of L. We can further relate this to thermodynamic derivatives by noting that L is typically linearly dependent on certain "fields" (*e.g.* pressure or magnetic field), with derivatives that correspond to measurable macroscopic properties (*e.g.* volume or magnetization). This linearity allows us to write the Fisher information even more simply: when $\langle \partial^2 L / \partial \mu^2 \rangle = 0$,

$$\mathcal{I}(\mu) = -\frac{\partial}{\partial \mu} \left\langle \frac{\partial L}{\partial \mu} \right\rangle. \tag{24}$$

(To see this, explicitly take the derivative of the expectation value:

$$\frac{\partial}{\partial \mu} \left\langle \frac{\partial L}{\partial \mu} \right\rangle = \frac{\partial}{\partial \mu} \left[Z^{-1} \sum_x \exp(-L(x)) \frac{\partial L(x)}{\partial \mu} \right] = -\left\langle \left(\frac{\partial L}{\partial \mu} \right)^2 \right\rangle + \left\langle \frac{\partial L}{\partial \mu} \right\rangle^2 + \left\langle \frac{\partial^2 L}{\partial \mu^2} \right\rangle, \tag{25}$$

which is equal to $-\mathcal{I}(\mu)$ from Eq. (23) when the last term is zero.)

Connecting this result to our equilibrium model, the susceptibility and specific heat are related to the Fisher information with respect to external field h_{ext} and temperature T (with units in which Boltzmann's constant $k_B = 1$):

$$\frac{\mathcal{I}(h_{\text{ext}})}{n} = \frac{1}{n}\frac{\partial\langle s\rangle}{\partial h_{\text{ext}}} = \chi. \tag{26}$$

$$\frac{\mathcal{I}(1/T)}{n} = \frac{T^2}{n}\frac{\partial\langle E\rangle}{\partial T} = T^2 C_s. \tag{27}$$

The amount of change in the entire distribution over fights is expressed in terms of a single "order parameter," the average fight size in the case of varying h_{ext} (and the average energy in the case of varying $1/T$). This implies that if one is trying to infer small changes in the external field h_{ext} by watching the composition of fights, one loses nothing by simply recording the fight sizes.

SUPPLEMENTARY REFERENCES

[1] Barton, J. & Cocco, S. Ising models for neural activity inferred via selective cluster expansion: structural and coding properties. *Journal of Statistical Mechanics: Theory and Experiment* **2013**, P03002 (2013).

[2] Tchernookov, M. & Nemenman, I. Predictive information in a nonequilibrium critical model. *J Stat Phys* **153**, 442 (2013).

[3] Prokopenko, M., Lizier, J. T., Obst, O. & Wang, X. R. Relating Fisher information to order parameters. *Physical Review E* **84**, 041116 (2011).

[4] Beggs, J. M. The criticality hypothesis: how local cortical networks might optimize information processing. *Philosophical transactions. Series A, Mathematical, physical, and engineering sciences* **366**, 329–43 (2008).

[5] Stanley, H. E. *Introduction to phase transitions and critical phenomena* (Oxford University Press, 1971).

[6] Mezard, M., Parisi, G. & Virasoro, M. *Spin Glass Theory And Beyond*, vol. 9 of *World Scientific Lecture Notes in Physics* (World Scientific, 1987).

[7] Georges, A. Strongly Correlated Electron Materials: Dynamical Mean-Field Theory and Electronic Structure. In Avella, A. & Mancini, F. (eds.) *Lectures on the Physics of Highly*

Correlated Electron Systems VIII: Eighth Training Course, vol. 3, 71 (American Institute of Physics, 2004). 0403123.

[8] Georges, A. & Yedidia, J. S. How to expand around mean-field theory using high-temperature expansions. *Journal of Physics A: Mathematical and General* **24**, 2173–2192 (1991).

[9] Cover, T. M. & Thomas, J. A. *Elements of Information Theory* (Wiley, 1991).

[10] Crooks, G. Measuring Thermodynamic Length. *Physical Review Letters* **99**, 100602 (2007).
